# Prognostic value of pneumococcal urinary antigen test in community-acquired pneumonia

**DOI:** 10.1371/journal.pone.0200620

**Published:** 2018-07-20

**Authors:** Byunghyun Kim, Joonghee Kim, You Hwan Jo, Jae Hyuk Lee, Ji Eun Hwang, Min Ji Park, Sihyung Lee

**Affiliations:** Department of Emergency Medicine, Seoul National University Bundang Hospital, Gyeonggi-do, Republic of Korea; Public Health England, UNITED KINGDOM

## Abstract

**Background:**

The pneumococcal urinary antigen test (UAT) has been known to improve sensitivity and specificity for the diagnosis of pneumococcal pneumonia. Associations of UAT results with prognosis in community acquired pneumonia (CAP) are not known. We hypothesized that positive UAT is associated with a good prognosis, and incorporation of UAT into CRB65 would improve its prognostic performance.

**Methods:**

In this registry-based retrospective study, we analyzed CAP patients over a 10-year period beginning in April 2008. Patients who had UAT results were included in multivariable extended Cox-regression analyses to determine the association between UAT positivity and 30-day mortality. UAT results were incorporated for patients with a CRB65 score of 1 by subtracting 1 from the scoring system if the test was positive. The performance of the modified scoring systems was assessed with area under the receiver operating characteristic (AUROC) curves.

**Results:**

Among 5145 CAP patients, total 2280 patients had UAT results and were included in analyses. A positive UAT result was associated with a good prognosis after a week of hospitalization (aHR, 0.14; p = 0.007). After modification of CRB65 using UAT results, positive and negative predictive values for 30-day mortality were increased from 7.7 to 8.3 (p<0.001) and 98.9 to 99.1 (p = 0.010). The AUROC increased from 0.73 to 0.75 (p<0.001).

**Conclusions:**

Positive results on UAT could be considered as a good prognostic factor in CAP. UAT could be used as a useful tool in deciding whether to refer patients to the hospital, especially in moderate CAP with a CRB score of 1.

## Introduction

Community acquired pneumonia (CAP) is a common disease but represents a major cause of mortality and morbidity, not to mention high cost. In Europe, the mortality rate from CAP ranges from <1% to 48%, depending on the country and patient characteristics [1]. These differences in mortality rates for patients led to the development of scoring systems that assess severity and predict mortality [2,3]. Especially the CURB65 or CRB65 scoring system from the British Thoracic Society (BTS) is widely used and there have been many modified versions to facilitate the decision making for the patient admission [2–5].

It is known that *Streptococcus pneumoniae* is the most common etiologic organism in CAP, overall representing 35% of isolated pathogen [6,7]. In addition to pneumococci, many other microorganisms are known to cause CAP and mortality rates differ for each species [6,7]. In particular, in the case of gram-negative bacteria (GNB), it is known that the mortality rate is higher than that of non-GNB pneumonia [8].

The pneumococcal urine antigen test (UAT) is an assay used to detect the C-polysaccharide antigen of *Streptococcus pneumoniae* in the urine of patients [9,10]. Advantages of the UAT are that collecting samples is relatively easy and its result is reported in real time (~15 min). Additionally, with moderate sensitivity (50–80%) and excellent specificity (>90%), the UAT increased the detection rate of pneumococcal pneumonia, regardless of antibiotic administration [11–13]. It was reported in a previous study that a positive UAT result is associated with poor outcomes in patients with pneumococcal pneumonia proven from blood cultures [11]. However, there are no studies investigating its prognostic implications in general CAP patients.

Therefore, the primary objective of this study was to investigate the prognostic implication of UAT results in general CAP patients. The secondary objective was to test whether incorporating UAT results to currently popular scoring systems (CRB65 and CURB65) improves their prognostic performance.

## Methods

### Study design and setting

This is a retrospective study analyzing a prospective registry of patients with pneumonia in the emergency department (ED) of a single tertiary academic hospital. The registry included the patient’s baseline characteristics and co-morbidities. Initial vital signs and laboratory results were included in the registry as well as the CURB65 and CRB65 scores for each patients. Additionally, the registry included any pathogens isolated from blood cultures or respiratory cultures performed in the ED, as well as UAT results. For the UAT, the Binax NOW immunochromatography method (Alere BinaxNow, Streptococcus pneumoniae Antigen Card; Alere Inc., Waltham, MA) was used in accordance with the manufacturer’s instructions. This study was performed at a 950-bed tertiary academic hospital with an annual ED census of 90,000 patients and Seoul National University Bundang Hospital IRB approved this study and waiver of informed consent.

### Study participants

We analyzed registry patients seen between April 2008 and March 2017. Patients were included if they were over age 18 when they visited the ED and were diagnosed with CAP, as defined by new radiographic findings on chest X-ray or chest CT with clinical symptoms that were suggestive of lower respiratory tract infection such as fever, cough or sputum production. Patients were excluded if they had one or more of the following criteria: (1) patients who met the diagnostic criteria for hospital-acquired pneumonia (HAP), health care-associated pneumonia (HCAP), or ventilator-associated pneumonia (VAP) [14], (2) patients with tuberculosis, HIV infection, or obstructive pneumonia due to cancer or other etiologies, (3) patients who were transferred to other hospital after the ED arrival.

### Statistical analysis

Categorical variables were reported using frequencies or proportions, whereas continuous variables were reported using the mean with standard deviation. Student’s t-test, Wilcoxon’s rank-sum test, the chi-squared test, or Fisher’s exact test were performed, as appropriate, for comparisons between the groups.

The association between UAT positivity and 30-day mortality was assessed using univariable and multivariable extended Cox-regression analysis. The proportional hazard assumption of the multivariable model was assessed by means of the analysis of Schoenfeld residuals. Variables with significant interactions with time were treated as time-varying coefficients, with different coefficients over two distinct periods as follows: early (within a week) and late (after a week). We constructed two multivariable models with different adjustment variable sets. In the first one, only the CURB65 score was included. In the second one, multiple covariates, selected based on Akaike’s information criterion, were included. The results are presented as hazard ratios (HRs) with 95% confidence intervals (CIs). The level of significance was set at p < 0.05.

To test the performance of each scoring system, receiver operating characteristic (ROC) curves were constructed, and the area under the curves (AUCs) were measured. To test the improvement of each scoring system with UAT results, AUCs, sensitivities, specificities, positive predictive values (PPVs) and negative predictive values (NPVs) for each scoring systems were compared before and after modification. The modifications to the current scoring systems were applied to intermediate risk patients with a CRB score of 1 or a CURB65 score of 2, where the determination for admission of patients can be ambiguous [15]. The modification method for each scoring system was as follows: (1) For the CURB65, patients with a CURB65 score of 2 had their score lowered to 1 if the UAT result was positive, (2) For the CRB65, patients with a CRB65 score of 1 had their score lowered to 0 if the UAT result was positive. Sensitivity analysis based on the cut off value for hospitalization was performed and compared before and after modification. All analyses were performed using STATA (version 13; StataCorp, College Station, TX) and R-packages, version 3.3.2 (R Foundation for Statistical Computing, Vienna, Austria).

## Results

During the study period, 7,931 patients were entered into the pneumonia registry. After excluding 2,786 patients who met the exclusion criteria, a total of 5,145 CAP patients were included in the study. Table 1 shows the clinical characteristics of patients with UAT tests compared to those without. UAT was performed more preferentially in patients with older age (65.3±17.7 vs. 63.3±18.6 years old), male sex (62.2% vs. 56.0%) and severe pneumonia with CRB65 score≥2 (23.2% vs. 16.9%). Clinical outcomes including 30-day mortality (5.7% vs. 3.9%) were poorer in the group that UAT was performed.

**Table 1:** Baseline characteristics of patients with or without UAT.

Category, Parameter	UAT performed (n = 2280)	UAT unperformed (n = 2865)	p value
**Epidemiological data**			
Mean age ± SD, years	65.3 ± 17.7	73.3 ± 18.6	<0.001
Male sex (%)	1417 (62.2)	1604 (56.0)	<0.001
Diabetes Mellitus (%)	494 (21.6)	498 (17.4)	<0.001
Hypertension (%)	836 (36.6)	949 (33.1)	0.008
Heart failure (%)	31 (1.3)	28 (1.0)	0.201
Cerebrovascular (%)	303 (13.3)	357 (12.5)	0.377
Renal failure (%)	134 (5.9)	149 (5.2)	0.290
Liver disease (%)	85 (3.7)	99 (3.5)	0.601
COPD (%)	345 (15.1)	271 (9.5)	<0.001
Known neoplasm (%)	356 (15.6)	373 (13.0)	0.008
**Vital signs, mean ± SD**			
Systolic blood pressure, mmHg	131 ± 24	132 ± 25	0.042
Diastolic blood pressure, mmHg	71 ± 15	73 ± 15	<0.001
Heart rate, beats/min	99 ± 19	97 ± 19	<0.001
Respiratory rate, cycles/min	21 ± 5	20 ± 4	<0.001
Body temperature, °C	37.5 ± 1.0	37.4 ± 1.0	0.246
**Laboratory findings, mean ± SD**			
WBC count, ×10^3^/mm^3^	11.6 ± 5.7	10.8 ± 5.2	<0.001
Hematocrit, %	37.8± 5.5	38.1 ± 5.3	0.018
Platelet count, ×10^3^/mm^3^	234.5 ± 102.4	226.1 ± 91.4	0.002
Sodium, mmol/dL	135.8 ± 4.8	136.4 ± 4.6	<0.001
Total CO_2_, mmol/dL	22.6 ± 3.0	23.0 ± 2.8	<0.001
Glucose, mg/dL	146 ± 75	136 ± 65	<0.001
Albumin, mg/dL	3.7 ± 0.5	3.8 ± 0.5	<0.001
BUN, mg/dL	19.6 ± 14.1	18.2 ± 14.4	<0.001
Creatinine, mg/dL	1.1 ± 0.9	1.0 ± 0.8	0.011
C-reactive protein, mg/dL	11.4 ± 8.8	8.7 ± 7.6	<0.001
Total Cholesterol, mg/dL	151.2 ± 42.6	156.3 ± 45.1	<0.001
Total Protein, mg/dL	6.7 ± 0.7	6.8 ± 1.4	<0.001
Prothrombin Time(INR)	1.1 ± 0.5	1.2 ± 1.9	0.540
**CRB65**			
**0**	686 (30.1)	1100 (38.4)	<0.001
**1**	1066 (46.7)	1282(44.7)	0.151
**2 or more**	528 (23.2)	483 (16.8)	<0.001
CURB65*			
0 or 1	1373 (60.4)	1878 (67.5)	<0.001
2	563 (24.4)	607 (21.8)	0.015
3 or more	339 (14.9)	295 (10.6)	<0.001
Time to 1^st^ antibiotics ± SD, min	202.1 ± 139.3	210.8 ± 134.2	0.064
Admission rate (%)	1357 (59.5)	932 (32.5)	<0.001
Ventilator rate (%)	186 (8.2)	140 (4.9)	<0.001
ICU admission rate (%)	212 (9.3)	152 (5.3)	<0.001
30-day mortality (%)	130 (5.7)	111 (3.9)	0.002

Abbreviations: SD, standard deviation; COPD, chronic obstructive pulmonary disease; WBC, white blood cell; BUN, blood urea nitrogen. Student’s t-test, Wilcoxon’s rank-sum test, the chi-squared test, or Fisher’s exact test were performed, as appropriate.

*90 patients without BUN results were not included in analysis

We selected patients with UAT results (N = 2,280) for our main analysis. Table 2 shows clinical characteristics of patients with positive test results (N = 229, 10.0%) compared to those with negative results (N = 2051, 90.0%). Patients with positive UAT had higher mean age (70.0±14.6 vs. 64.7±17.9 years old), lower systolic blood pressure (131±24 vs. 126±27mmHg), and diastolic blood pressure (71±14 vs. 68±15mmHg), higher respiratory rate (21±5 vs. 22±6/min), and more severe laboratory abnormalities including BUN (23.0±16.2 vs. 19.2±13.8). The group with positive UAT suffered more severe pneumonia with CRB65≥2 (32.9% vs. 18.3%). While significantly higher proportion of the patients were admitted in the group, overall ventilator care, ICU admission and 30-day mortality rates were not significantly different.

**Table 2:** Baseline characteristics according to UAT results.

Category, Parameter	UAT positive (n = 229)	UAT negative (n = 2051)	p value
**Epidemiological data**			
Mean age ± SD, years	70.0 ± 14.6	64.7 ± 17.9	<0.001
Male sex (%)	152 (66.4)	1265 (61.7)	0.056
Diabetes Mellitus (%)	42 (18.3)	452 (22.0)	0.198
Hypertension (%)	88 (38.4)	748 (36.4)	0.560
Heart failure (%)	3 (1.3)	28 (1.4)	1.000
Cerebrovascular (%)	34 (14.8)	269 (13.1)	0.464
Renal failure (%)	14 (6.1)	120 (5.8)	0.873
Liver disease (%)	10 (4.4)	75 (3.7)	0.591
COPD (%)	36 (15.7)	309 (15.1)	0.793
Known neoplasm (%)	37 (16.2)	319 (15.6)	0.811
**Vital signs, mean ± SD**			
Systolic blood pressure, mmHg	126 ± 27	131 ± 24	0.006
Diastolic blood pressure, mmHg	68 ± 15	71 ± 14	0.001
Heart rate, beats/min	101 ± 22	99 ± 19	0.339
Respiratory rate, cycles/min	22 ± 6	21 ± 5	0.042
Body temperature, °C	37.5 ± 1.1	37.5 ± 1.0	0.285
**Laboratory findings, mean ± SD**			
WBC count, ×10^3^/mm^3^	12.9 ± 6.5	11.5 ± 5.6	0.002
Hematocrit, %	37.7 ± 5.7	37.8 ± 5.5	0.804
Platelet count, ×10^3^/mm^3^	226.2 ± 103.7	235.4 ± 102.2	0.202
Sodium, mmol/dL	135.1 ± 5.6	135.8 ± 4.7	0.077
Total CO_2_, mmol/dL	22.2 ± 3.1	22.6 ± 3.0	0.031
Glucose, mg/dL	151 ± 103	146 ± 71	0.440
Albumin, mg/dL	3.6 ± 0.6	3.7 ± 0.5	0.015
BUN, mg/dL	23.0 ± 16.2	19.2 ± 13.8	<0.001
Creatinine, mg/dL	1.1 ± 0.8	1.0 ± 0.9	0.379
C-reactive protein, mg/dL	13.6 ± 9.6	11.2 ± 8.7	<0.001
Total Cholesterol, mg/dL	141.6 ± 41.9	152.3 ± 42.5	<0.001
Total Protein, mg/dL	6.5 ± 0.9	6.7 ± 0.7	<0.001
Prothrombin Time (INR)	1.2 ± 0.4	1.1 ± 0.5	0.365
**CRB65 (%)**			
** 0**	41 (17.9)	645 (31.4)	<0.001
** 1**	113 (49.3)	953 (46.5)	0.407
** 2 or more**	75 (32.9)	453 (18.3)	<0.001
CURB65* (%)			
** 0 or 1**	108 (47.4)	1265 (62.8)	<0.001
** 2**	64 (28.1)	499 (24.4)	0.220
** 3 or more**	56 (24.6)	283 (13.8)	<0.001
Time to 1^st^ antibiotics ± SD, min	189.8 ± 147.6	203.6 ± 138.3	0.165
Admission rate (%)	167 (72.9)	1190 (58.0)	<0.001
Ventilator rate (%)	23 (10.0)	163 (8.0)	0.272
ICU admission rate (%)	25 (11.0)	187 (9.1)	0.357
30-day mortality (%)	9 (3.9)	121 (5.9)	0.223

Abbreviations: UAT streptococcal urinary antigen test; SD, standard deviation; COPD, chronic obstructive pulmonary disease; WBC, white blood cell; BUN, blood urea nitrogen. Student’s t-test, Wilcoxon’s rank-sum test, the chi-squared test, or Fisher’s exact test were performed, as appropriate.

*5 patients without BUN results were not included in analysis.

**Fig 1:**
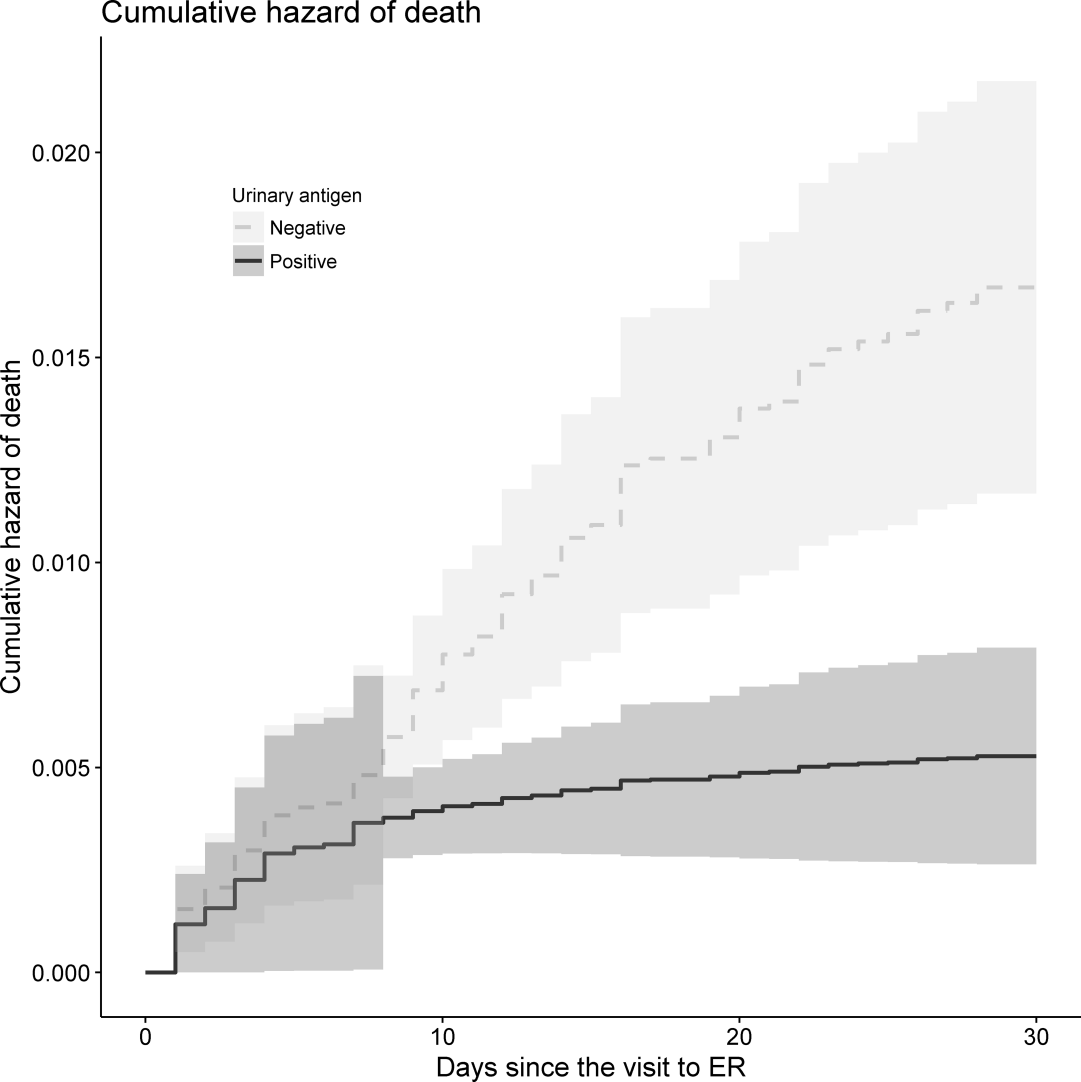
Cumulative hazards according to UAT results.

Table 3 shows the results of univariable and multivariable extended Cox-regression model for death in earlier (< 1 week) and later (> 1 week) period of the one-month observation. Mortality risk was not significantly different between patients with positive and negative UAT result in earlier period (HR, 1.3; 95% CI, 0.59–2.87; p = 0.521). However, the risk was significantly lower in positive UAT group in the later period (HR, 0.24; 95% CI, 0.06–0.98; p = 0.047). The association was still significant after multivariable adjustment. Seven days after visiting the ED, mortality risk was significantly lower in patients with positive UAT (HR, 0.14; 95% CI, 0.03–0.58; p = 0.007). Fig 1 visualizes the cumulative hazards according to UAT result.

**Table 3:** Univariable and multivariable extended Cox-regression analyses.

**Univariate analysis**	**Hazard ratio (95% CI), p value**	
	**Early**	**Late**
UAT positive	1.30 (0.59–2.87), 0.521	0.24 (0.06–0.98), 0.047
**Multivariate analysis**	**Hazard ratio (95% CI), p value**	
	**Early**	**Late**
UAT positive	0.76 (0.33–1.75), 0.517	0.14 (0.03–0.58), 0.007
Sex	1.06 (0.59–1.92), 0.842	1.89 (1.06–3.36), 0.032
Cerebrovascular disease	0.92 (0.48–1.78), 0.813	2.00 (1.19–3.38), 0.009
Initial body temperature	0.59 (0.43–0.81), 0.001	0.84 (0.66–1.08), 0.180
CURB65	1.72 (1.30–2.27), <0.001	0.88 (0.68–1.12), 0.296
Ventilator apply	1.96 (1.10–3.51), 0.023	7.34 (4.54–11.89), <0.001
Age	1.03 (1.01–1.05), <0.001	
Known neoplasm	2.32 (1.58–3.41), <0.001	
COPD	0.50 (0.28–0.88), 0.016	
Initial purse rate	1.01 (1.00–1.02), 0.002	
Initial respiratory rate	1.06 (1.04–1.09), <0.001	
Cholesterol level	1.01 (1.00–1.01), <0.001	
Albumin level	0.23 (0.16–0.33), <0.001	

Abbreviations: UAT streptococcal urinary antigen test; COPD, chronic obstructive pulmonary disease.

### Performance of CRB65 and CURB65 after modification

Both CRB65 and CURB65 score were modified to subtract 1 if UAT was positive when the scores indicate intermediate risk (CRB65 score 1 and CURB65 score 2). Table 4 shows the performances of CRB65 and CURB65 scores before and after the modification in prediction of 30-day mortality. In CRB65, specificity, PPV and NPV were significantly increased (31.6 to 36.8%, 7.7 to 8.3% and 98.9 to 99.1%, respectively). In CURB65, the modification resulted in a statistically significant increase in the specificity and PPV (62.7 to 65.6% and 11.2 to 11.9%, respectively).

**Table 4:** Sensitivity, specificity, PPV and NPV changes from modification of scoring systems.

	30d mortality (%)	UAT positive (%)	Cut-off	Sensitivity	Specificity	PPV	NPV
**CRB65**							
**0**	**7/686 (1.0)**	41/686 (6.0)					
**1**	**50/1066 (4.7)**	113/1066 (10.6)	**1**	**94.6**	**31.6**	**7.7**	**98.9**
**2**	48/435 (11.0)	60/435 (13.8)					
**3**	22/81 (27.2)	12/81 (14.8)					
**4**	3/12 (25.0)	3/12 (25.0)					
CRB65P*							
**0**	**7/799 (0.9)**	154/799 (19.3)					
**1**	**50/953 (5.3)**	0/953 (0)	**1**	**94.6**	**36.8**	**8.3**	**99.1**
**2**	48/435 (11.0)	60/435 (13.8)					
**3**	22/81 (27.2)	12/81 (14.8)					
**4**	3/12 (25.0)	3/12 (25.0)					
***P* value**				**NA**	**<0.001**	**<0.001**	**0.010**
**CURB65**							
**0**	4/602 (0.7)	32/602 (5.3)					
**1**	**25/771 (3.2)**	76/771 (9.9)					
**2**	**42/563 (7.5)**	64/563 (11.4)	**2**	**77.7**	**62.7**	**11.2**	**97.8**
**3**	37/268 (13.8)	44/268 (16.4)					
**4**	19/61 (31.2)	9/61 (14.8)					
**5**	3/10 (30.0)	3/10 (30.0)					
CURB65P*							
**0**	4/602 (0.7)	32/602 (5.3)					
**1**	**26/835 (3.1)**	140/835 (16.8)					
**2**	**41/499 (8.2)**	0/499 (0)	**2**	**76.9**	**65.6**	**11.9**	**97.9**
**3**	37/268 (13.8)	44/268 (16.4)					
**4**	19/61 (31.2)	9/61 (14.8)					
**5**	3/10 (30.0)	3/10 (30.0)					
***P* value**				**0.317**	**<0.001**	**<0.001**	**0.731**

Sensitivity and specificity were compared using McNemar test. PPV and NPV were compared using a generalized score statistic. Abbreviations: PPV positive predictive value; NPV, negative predictive value.

*modified CRB65 or CURB65 using the streptococcal urinary antigen test.

**Fig 2:**
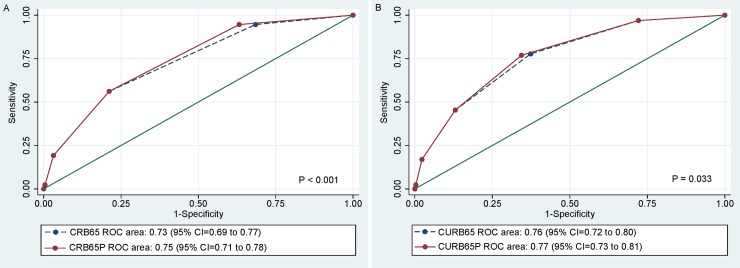
Comparison of AUCs for scoring systems before and after modification. (A) CRB65 (before) and CRB65P (after), (B) CURB65 (before) and CURB65P (after).

Fig 2 shows the AUCs for each scoring system before and after the modification. The inclusion of UAT to CRB65 resulted in increase of its AUC from 0.73 (95% CI, 0.69–0.77) to 0.75 (95% CI, 0.71–0.78) with statistical significance (p<0.001). Similarly, it increased the AUC of CURB65 form 0.76 (95% CI, 0.72–0.80) to 0.77 (95% CI, 0.73–0.81) (p = 0.033).

## Discussion

According to National Institute for Health and Care Excellence (NICE) guideline, the latest CAP guideline, the CRB65 is recommended for primary care settings and CURB65 in hospital settings [15]. CRB65 is a useful tool in small clinics for determining whether to refer patients to hospitals because it requires no laboratory tests [3]. A score of more than 1 point on the CRB65, which is the cut-off point for hospital referral, has high sensitivity but low specificity, and it seems many patients are unnecessarily referred to the hospital. In addition, the guidelines do not provide specific hospitalization criteria for patients with a CRB65 score of 1. The results of this study show relatively simple UAT, not requiring venipuncture, can help determine hospital referral for patients with ambiguous referral criteria. Among 1,066 patients with a CRB65 score of 1, 113 patients had positive UAT results and their 30-day mortality rate was 0% (Table 4). Similarly, of 563 patients with a CURB65 score of 2, 64 had a positive UAT result and their 30-day mortality rate was 1.5%, which was lower than those patients with a CURB65 score of 1.

In addition to helping clarify the criteria for hospital referral, the UAT can also help to select targeted antibiotics for identified pathogens [16,17]. According to the guidelines, dual antibiotics, consisting of amoxicillin and a macrolide, are preferred for patients with moderate severity CAP [2,15]. If patients with a CRB65 score of 1 are positive on the UAT, the dual antibiotics could be de-escalated to single amoxicillin regimen. There are controversial studies on the modification of antibiotics according to UAT results [12,13,18,19]. On the pro side, targeting antibiotics based on UAT results decreases antibiotic resistance and costs by reducing unnecessary antibiotics, with a low relapse rate of less than 5% [13,18]. On the other hand, opponents say there is no difference in side effects, but the relapse rate is increasing [19]. However, the study by Falguera et al. was based on small numbers and only included hospitalized patients.

The relationship between UAT results and mortality has been studied only in patients with bacteremic pneumococcal pneumonia proven by blood culture [11]. In the study by Zalacain et al., the positive UAT result was associated with increased ICU admission, treatment failure and adverse outcomes in hospitalized patients. This was also observed in our study, where patients with positive UAT suffered more severe pneumonia. However, this differences in hospital courses were preceded by significantly different initial CRB65 and CURB65 scores. It has been unknown how to interpret the test results within context of fixed initial CRB65 and CURB65. In our study, the positive UAT was associated with lower risk of mortality after adjustment for CURB65 score and other significant covariates. This suggests if initial presentation is similar, UAT result can have opposite meaning.

We can summarize our interpretation of this reversed association as follows. The worse initial presentation and hospital courses in patients with positive UAT could be due to higher with bacterial burden and inflammation [20]. However, if their initial presentation is not severe even with positive UAT, it is possible that the burdens of the disease are not so different from their counterparts in patients with negative UAT. In this case, the main determinant of their mortality would be responsiveness to the antibiotic treatment. We think once appropriate antibiotics is administered to the patients with positive UAT, which is highly probable because most empirical antibiotic regimens for CAP cover pneumococcus, their hospital courses would be better compared to their counterparts. Patients with a negative UAT result may have more chance to have inappropriate antibiotics until culture results were reported and even may continue to receive initial empirical antibiotics without knowing the causative organism. It is possible this delay in organism specific treatment may have resulted in the difference of risk of mortality in the later period of the patients’ courses. However, we did not factor in the appropriateness of antibiotic regimen in our analyses because there would be a lot of cases with negative UAT cases whose causative organism is indeterminate or unknown. Also it should be mentioned that it is possible the time to initial antibiotics administration could be contributing factor. However, the time to initial antibiotics were not significantly different UAT positive and negative group in this study (Table 2). Another possible explanation for the difference in the patient course could be the difference in pathogenesis between GNB and Gram-positive bacteria (GPB) [21,22]. In the previous studies, GNB were detected in more severe sepsis with multi-organ failure versus GPB. And the mortality rate of pneumococcal pneumonia was lower than pneumonia due to unidentified pathogens or due to other GNBs [3,6]. Multi-organ failure with severe inflammation and delayed immune paralysis could result in mortality differences in the late period [23].

This retrospective study has several limitations. First, we could not show clinically significant shortening of antibiotic administration time using the advantage of UAT reported in real time. Most patients received empirical antibiotics as a routine practice, regardless of UAT results, and most results of UAT were confirmed later because of laboratory delay in our facility. Second, significantly different baseline characteristics and mortality rates between patients with and without UAT results could create a selection bias (Table 1). This may be a limitation, but it can be considered to reflect real-world hospital situations. Third, we could not show the percentage of patients with pneumococcal vaccine. Since 2013, the National Immunization Program provides free 23-valent pneumococcal polysaccharide vaccine (PPSV23) for all people aged 65 years or older in South Korea and the vaccination rate has reached 60%, as of May 2017 [24]. As the vaccination rate increases, the effect of UAT results as a good prognostic factor will become more prominent. Finally, we excluded patients classified with HCAP, such as nursing home residents. Although studies in which the CRB65 and CURB65 were derived, excluded nursing home residents, recent guidelines consider nursing home residents as CAP patients and the concept of HCAP is being minimized [15,25].

In conclusion, a positive result on the pneumococcal UAT can be considered as a good prognostic factor in CAP patients if their initial presentation is similar. In addition, incorporating UAT to current CRB65 and CURB65 score can improve their predictive performances. Further research is needed, including multicenter studies or group-based studies.
